# Self-Selection of Bathroom-Assistive Technology: Development of an Electronic Decision Support System (Hygiene 2.0)

**DOI:** 10.2196/16175

**Published:** 2020-08-10

**Authors:** Manon Guay, Karine Latulippe, Claudine Auger, Dominique Giroux, Noémie Séguin-Tremblay, Josée Gauthier, Catherine Genest, Ernesto Morales, Claude Vincent

**Affiliations:** 1 School of Rehabilitation Université de Sherbrooke Sherbrooke, QC Canada; 2 Center for Research on Aging Sherbrooke, QC Canada; 3 Department of Teaching and Learning Studies Université Laval Quebec, QC Canada; 4 School of Rehabilitation Faculty of Medicine Université de Montréal Montreal, QC Canada; 5 Centre for Interdisciplinary Research in Rehabilitation of Greater Montreal Montreal, QC Canada; 6 Center of Excellence on Aging Quebec Quebec, QC Canada; 7 Department of Rehabilitation Faculty of Medicine Université Laval Quebec, QC Canada; 8 VITAM - Centre de recherche en santé durable Quebec, QC Canada; 9 CIUSSS de la Mauricie-et-du-Centre-du-Québec Trois-Rivières, QC Canada; 10 CIUSSS de la Capitale-Nationale Quebec, QC Canada; 11 Center for Interdisciplinary Research in Rehabilitation and Social Integration Quebec, QC Canada

**Keywords:** hygiene, activities of daily living, decision aids, occupational therapy, aging, self-help devices, universal design, accidental falls, mobile phone

## Abstract

**Background:**

A clinical algorithm (Algo) in paper form is used in Quebec, Canada, to allow health care workers other than occupational therapists (OTs) to make bathroom adaptation recommendations for older adults. An integrated knowledge transfer process around Algo suggested an electronic version of this decision support system (electronic decision support system [e-DSS]) to be used by older adults and their caregivers in search of information and solutions for their autonomy and safety in the bathroom.

**Objective:**

This study aims to (1) create an e-DSS for the self-selection of bathroom-assistive technology by community-dwelling older adults and their caregivers and (2) assess usability with lay users and experts to improve the design accordingly.

**Methods:**

On the basis of a user-centered design approach, the process started with content identification for the prototype through 7 semistructured interviews with key informants of various backgrounds (health care providers, assistive technology providers, and community services) and 4 focus groups (2 with older adults and 2 with caregivers). A thematic content transcript analysis was carried out and used during the creation of the prototype. The prototype was refined iteratively using think-aloud and observation methods with a clinical expert (n=1), researchers (n=3), OTs (n=3), older adults (n=3), and caregivers (n=3), who provided information on the usability of the e-DSS.

**Results:**

Overall, 4 themes served as the criteria for the prototype of the electronic Algo (Hygiene 2.0 [H_2_.0]): focus (safety, confidentiality, well-being, and autonomy), engage, facilitate (simplify, clarify, and illustrate), and access. For example, users first pay attention to the images (engage and illustrate) that can be used to depict safe postures (safety), illustrate questions embedded in the decision support tool (clarify and illustrate), and demonstrate the context of the use of assistive technology (safety and clarify).

**Conclusions:**

The user-centered design of H_2_.0 allowed the cocreation of an e-DSS in the form of a website, in line with the needs of community-dwelling older adults and their caregivers seeking bathroom-assistive technology that enables personal hygiene. Each iteration improved usability and brought more insight into the users’ realities, tailoring the e-DSS to the implementation context.

## Introduction

### Background

Performing personal hygiene is an essential daily activity for health and dignity that commonly becomes difficult with aging. A survey of 28,406 noninstitutionalized Canadians (50-104 years old) revealed that the prevalence of disability increased with age, exponentially increasing when considering the oldest old [1]. At 90 years of age, 21% of Canadians reported requiring assistance to wash themselves [1]. In such cases, adapting the bathroom environment with assistive technologies, such as bath seats, grab bars, or nonslip mats, is a common recommendation to promote autonomy and safety [2,3].

To address this issue in Quebec, a mainly French-speaking province of Canada, the clinical *algo*rithm Algo [4] has been proposed allowing occupational therapists (OTs) to collaborate with non-OTs: health care workers other than OTs, such as home health aides, social workers, and nurse assistants. In a situation deemed straightforward (ie, clients of standard morphology with predictable occupational performance in bath transfer in their standard shower stall or bathtub) [5], Algo supports decision making for home care clients who need recommendations on full-body hygiene, considering their preferences as well as their abilities and the actual physical environment in which they live [6].

**Figure 1:**
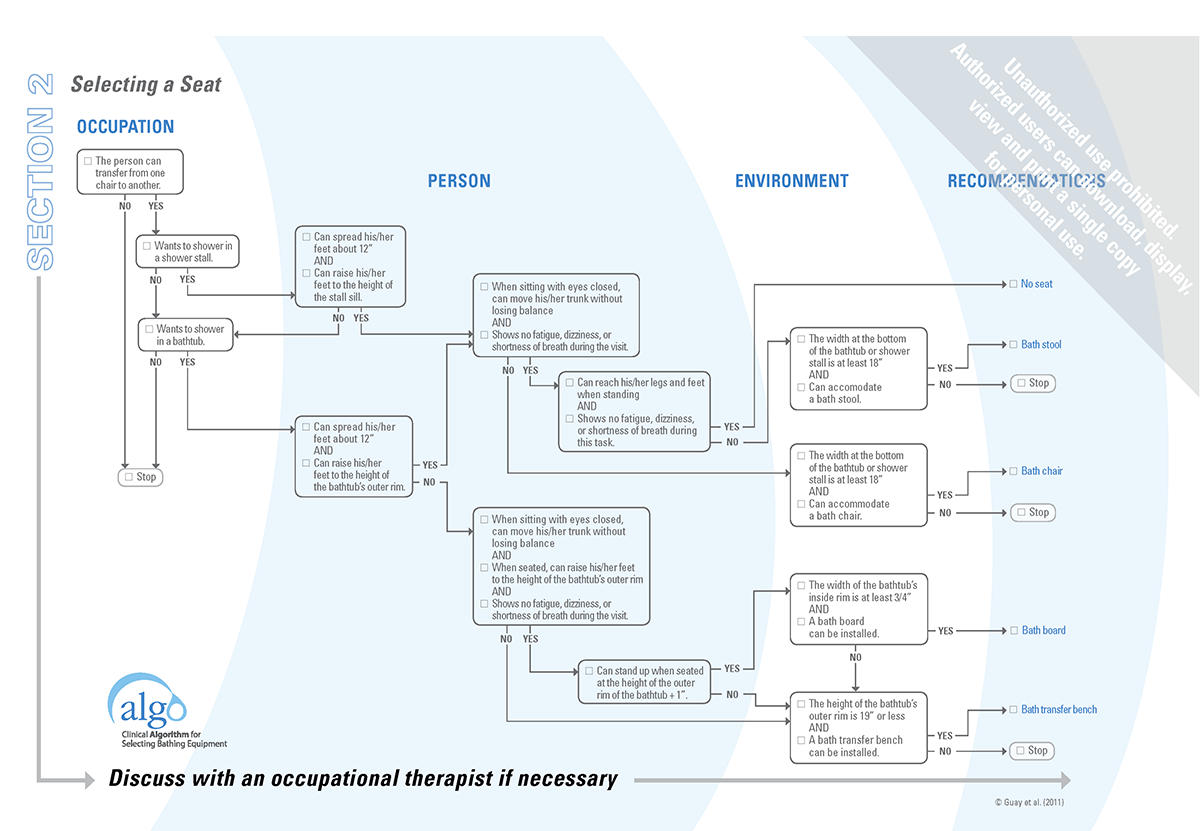
Section 2 of the paper format Algo.

Indeed, Algo is a clinical algorithm in paper form paired with a user guide that outlines the logical steps for non-OTs to select bathing equipment (Figure 1). It also identifies complex cases to be referred to an OT [6]. Older adults receive 1 of 9 possible recommendations [6], with or without assistive technology, specific to their bathing situation (ie, standing without a seat in the bathtub, sitting on a bath stool in the shower stall, or stop and refer to an OT). The content of the Algo was developed through an integrated knowledge approach [6,7], and psychometric studies [8-12] revealed, for example, that it guided non-OTs toward a bath seat that meets the needs of community-dwelling older adults in the majority of cases (mean 84%, SD 9%) [9]. The appropriateness rate of seats recommended by non-OTs did not statistically differ from that of 2 OTs [9].

In 2015, approximately half (48%) of the targeted end users knew about Algo, with half of these (24%) reporting that they had begun the implantation process in their clinical settings [13]. Since then, community OTs have ideated about converting the knowledge embedded in Algo into an electronic version to be used by their clients [14]. It appears to these OTs that Algo could be the foundation for an electronic decision support system (e-DSS) to help people make informed decisions about assistive technologies [15]. Usually, e-DSSs include first, a knowledge base; second, a program to combine that knowledge with user-specific information; and third, an interface used to collect data about the user and to provide the user with relevant information [16].

### Objectives

This study aims to adapt Algo’s paper version to an e-DSS for older adults and caregivers experiencing difficulty when performing personal hygiene. Specifically, the objectives were to (1) create an e-DSS for the self-selection of bathroom-assistive technology by community-dwelling older adults and their caregivers, and (2) assess usability with lay users and experts to improve the design accordingly.

## Methods

### Design Process

**Figure 2:**
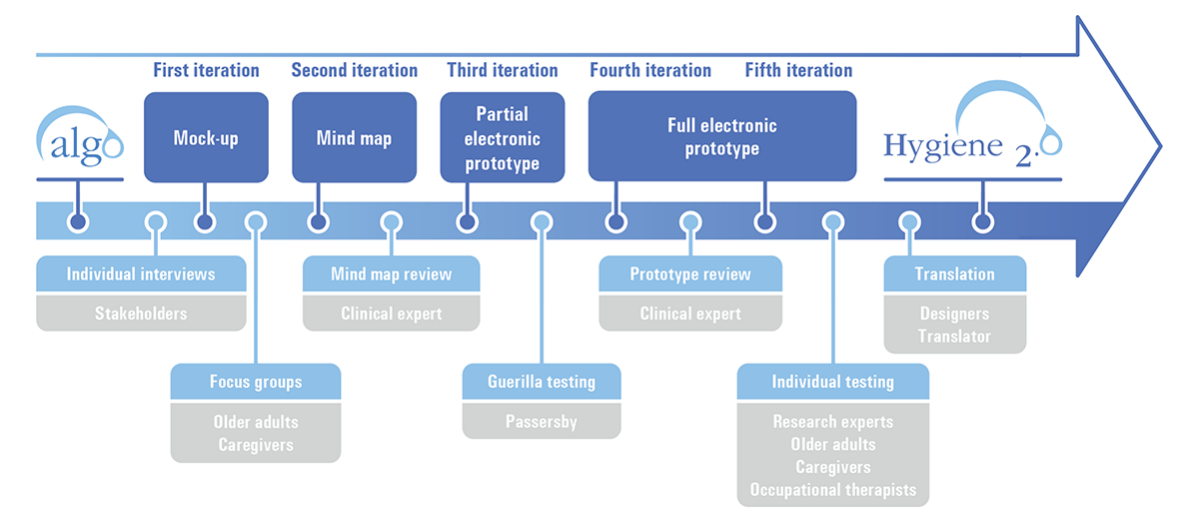
Iterative design process.

A user-centered design method [17] was conducted, in which an ongoing iterative process facilitated dialogue between potential users and designers (Figure 2). The designers are the principal investigator (an OT) and a research professional (background in mechanical engineering and cognitive ergonomics). The term older adults refers to community-dwelling adults aged at least 65 years who have difficulty completing their personal hygiene routine and their caregivers refers to people concerned about or assisting a community-dwelling older adult with their personal hygiene. Although Algo is the name of the algorithm in paper form, Hygiene 2.0 (H_2_.0) is the name given to the electronic version of the decision support system.

### First Iteration

In addition to the knowledge gathered during our previous studies, 7 semistructured interviews were conducted with stakeholders, recruited by word-of-mouth, to participate in the research project for their professional experience by providing information on bathing assistive technology for the elderly or selecting and providing such technology. The interviews aimed at gaining deeper knowledge of the needs an e-DSS should address in their work context. In a private room in their work settings, participants were encouraged to express their point of view following open-ended questions from an interview guide developed by the research team and iteratively modified (Multimedia Appendix 1). Interviews were recorded and transcribed. Qualitative data analysis principles of thematic data condensation were first conducted by the interviewer using Microsoft Word and Excel software. A total of 2 members of the research team reviewed and clarified the theme definitions by analyzing the transcription extracts iteratively (individual coding and dyad work sessions). Memos were used to facilitate reflexivity and research team discussions.

**Figure 3:**
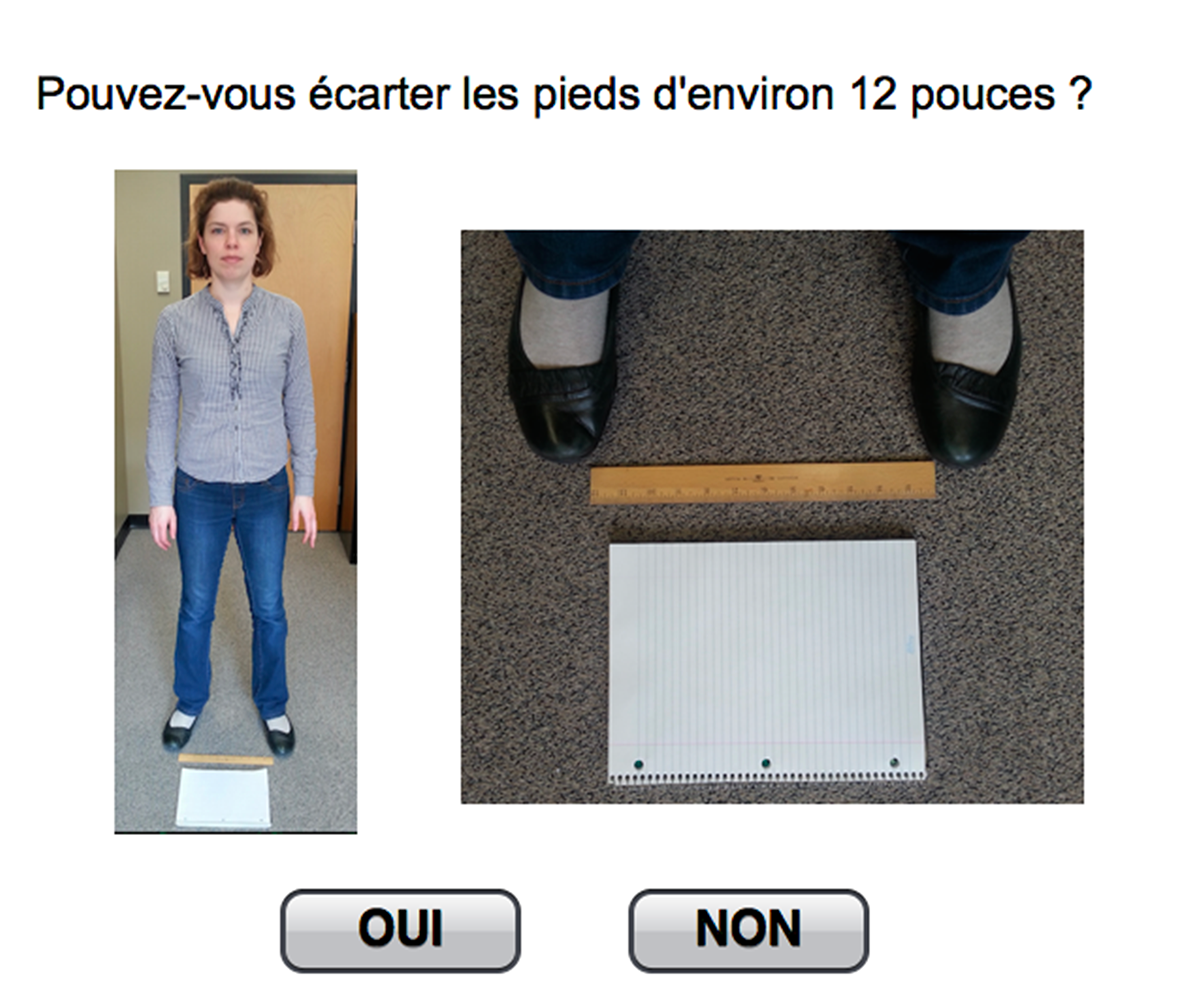
Excerpt from the PowerPoint mock-up (translation: Can you spread your feet about 12 inches? Options: Yes, No).

The designers considered these themes while creating the first version of an electronic Algo as they represent user needs related to this e-DSS. A mock-up was designed on PowerPoint as a rough draft using 6 questions extracted from Algo and pictures taken by the designers to illustrate them (Figure 3). The mock-up allowed the collection of participants’ spontaneous comments and questions regarding specific features, facilitating the iterative design process.

A total of 4 focus groups were organized to give users the opportunity to express their experience regarding the choice of assistive technologies, their needs regarding health information, their use of computer technology, and their opinion on the PowerPoint mock-up (Multimedia Appendix 2). A moderator (the research professional), a content expert (the principal investigator), and a graduate student responsible for logistics were present in the room ensuring privacy.

Overall, 2 of those focus groups included older adults and were constituted with a systematic sampling procedure. Every fifth volunteer aged 65 years on a list from the Research Center on Aging was contacted. A research assistant reviewed with the volunteer, over the phone, the following inclusion criteria: having experienced difficulties with bathing per the definition of the *Functional Autonomy Measurement System* [18]. The exclusion criteria were cognitive impairment limiting expression or comprehension and inability to speak French.

Additionally, 2 focus groups were conducted with caregivers. Recruitment was performed in collaboration with 2 community resources (a domestic help service and a volunteer bureau), which helped identify caregivers for people having difficulty with bathing. The same exclusion criteria applied to older adults in the previous focus groups were applied to the caregivers.

All 4 focus groups were transcribed verbatim and analyzed with the methodology described above for the interviews, but using NVivo software (QSR International) to analyze the data. Modifications were made to the PowerPoint mock-up after each focus group. A grid relating the themes to specific solutions for the prototype was implemented to verify that each general theme emerging through the coding would be translated into practical solutions and that each modification to the prototype corresponded to the previously established themes (Multimedia Appendix 3). In addition to considering the user needs and context, tracking and correcting usability challenges were also considered within the iterative evolution of the prototype. Emerging knowledge was integrated in an ongoing process into the different versions of the prototypes.

### Second Iteration

The analysis of the focus groups integrated with the results from the interviews revealed that an offline mobile app and a responsive website would be the preferred formats for users of the adapted Algo. To do so, the Algo algorithm was broken down and reorganized into a diagram, referred to as a mind map [19], considering previously gathered data regarding the users and the context of use. For example, the item order in the paper form of Algo was modified to make the e-DSS easy to understand and minimize the steps before obtaining an assistive technology recommendation.

An OT with 25 years of clinical experience in home care for older adults was hired to conduct a thorough review of the mind map. She was familiar with Algo in paper form and had trained OTs and non-OTs on using the same. The expert conducted a careful reading of the results of the qualitative analysis and the mind map prototype. She was asked to provide her professional opinions on equivalency, noting missing or superfluous information, as well as on literacy (target audience: 10-year-old reader). A total of five 2-hour unstructured interviews were conducted with her (for a total of 10 hours), encouraging the think-aloud process and modifying the mind map live with her throughout the meetings.

### Third Iteration

The conception of a partial electronic prototype was initiated with the involvement of a programmer. The questions of the mind map relating to the recommendation “sitting on a bath stool in the shower stall” were programmed.

In parallel, to identify the best practices in web design for older adults, a literature review was conducted with the assistance of 2 librarians. The databases Education Resources Information Center (ERIC), AgeLine, Medical Literature Analysis and Retrieval System Online (MEDLINE), and Cumulated Index to Nursing and Allied Health Literature (CINAHL) were searched for publications before January 1, 2014, in French or English with such keywords as Internet*, health*, health education*, instructional design*, product design*, and older computer users*. Of the 47 references selected by the principal investigator for their relevance to the subject, the guidelines from Chaffin and Maddux [20], Czaja [21], Fisk et al [22], Nielsen [23], the National Institute on Aging (NIA) [24], and norms International Organization for Standardization (ISO) 9241-210 [25] and ISO 9241-11 [26] were applicable to this study. Relevant publications regarding literacy and usability published since then were also considered during the design: US Federal Plain Language Guidelines [27], a guide for communicating in the field of health from the Montreal Health and Social Services Agency [28], and a guide from the US Department of Health on the design of easy-to-use health websites [29].

**Figure 4:**
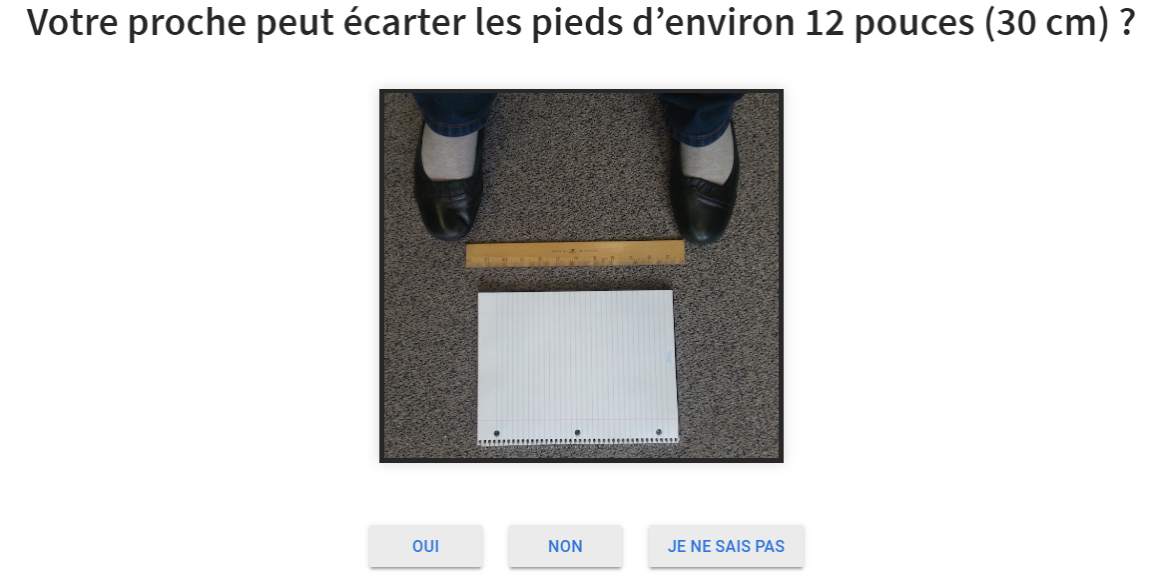
Excerpt from the partial prototype (translation: Can your family member or loved one stand with his or her feet about 12 inches [30 cm] apart? Options: Yes, No, I Don’t Know).

The partial electronic prototype (Figure 4) was used for guerrilla testing, which consists of validating a design by conducting quick usability tests with passersby in a public space [30]. In our case, this was done during the poster sessions of 2 geriatric and rehabilitation scientific conferences in Sherbrooke and Montreal, Canada. Volunteers were recruited among attendees (interdisciplinary Masters or PhD students, rehabilitation researchers, research professionals, health care professionals, and decision makers). Although these settings did not include older adults, they did include other stakeholders and offered a good opportunity to rapidly get feedback on the preliminary design. Specifically, these tests aimed at verifying the font and size of the text, the page layout, the understanding of questions and associated images, and the swift functioning of the navigation. After giving them minimal contextual setting, the programmer and a designer, respectively, observed and took note of the volunteers’ comments and behavior while they tested the prototype presented on a tablet. As the partial prototype was pared down and the testing method was quick (approximately 5 min), the comments were brief. The programmer and designer discussed these comments and notes were taken. Aspects with the greatest impact on usability (eg, loading of the web pages was slow, hyperlinks were not understood as well as buttons) were considered to make corresponding changes. When the comments were contradictory between volunteers and no trend could be identified, they were kept to be verified during the following tests.

### Fourth Iteration

A full prototype was programmed, with the process being performed backward, meaning each of Algo’s 9 potential recommendations was programmed and added to the prototype one at a time. After every addition, the designers reviewed the prototype independently, navigating systematically and randomly, to identify potential errors and suggest improvements. They compared the equivalence between questions within the original paper form of Algo and the prototype. Every discrepancy was noted and discussed among the research team to verify the rationale for changes within the results.

This procedure was repeated with a clinical OT; the expert hired for the second iteration was recruited. She had to independently compare the algorithm structure of the paper form and the electronic form and question discrepancies while thinking aloud. The interview lasted 134 min. Designers gathered observations in person, verifying that they had a rationale within the study results, iteratively modifying the grid relating the themes to specific solutions for the prototype. Incoherencies were discussed among designers and the prototype was modified accordingly to have it ready for usability testing with potential users. As these users advised during previous iterations, a professional photographer was hired, and 3 older adults were recruited by word-of-mouth to appear in pictures depicting the questions in the prototype.

### Fifth Iteration

The prototype was first tested with 3 researchers, recruited by word-of-mouth, having previously worked as clinical OTs. Although they were not the target users, they had clinical experience, and as researchers tend to be very thorough, these tests acted as a comprehensive review of the prototype. Moreover, 2 categories of users and 1 category of stakeholders were then tested: older adults, caregivers, and OTs. A sample of 3 participants from each category was formed as it is the most resource-efficient way to conduct testing [31]. Recruitment of the older adults and caregivers followed the same procedure described for the focus groups. Tests were conducted at each participant’s home. Overall, 3 OTs were recruited by word-of-mouth and tested in their work settings.

**Figure 5:**
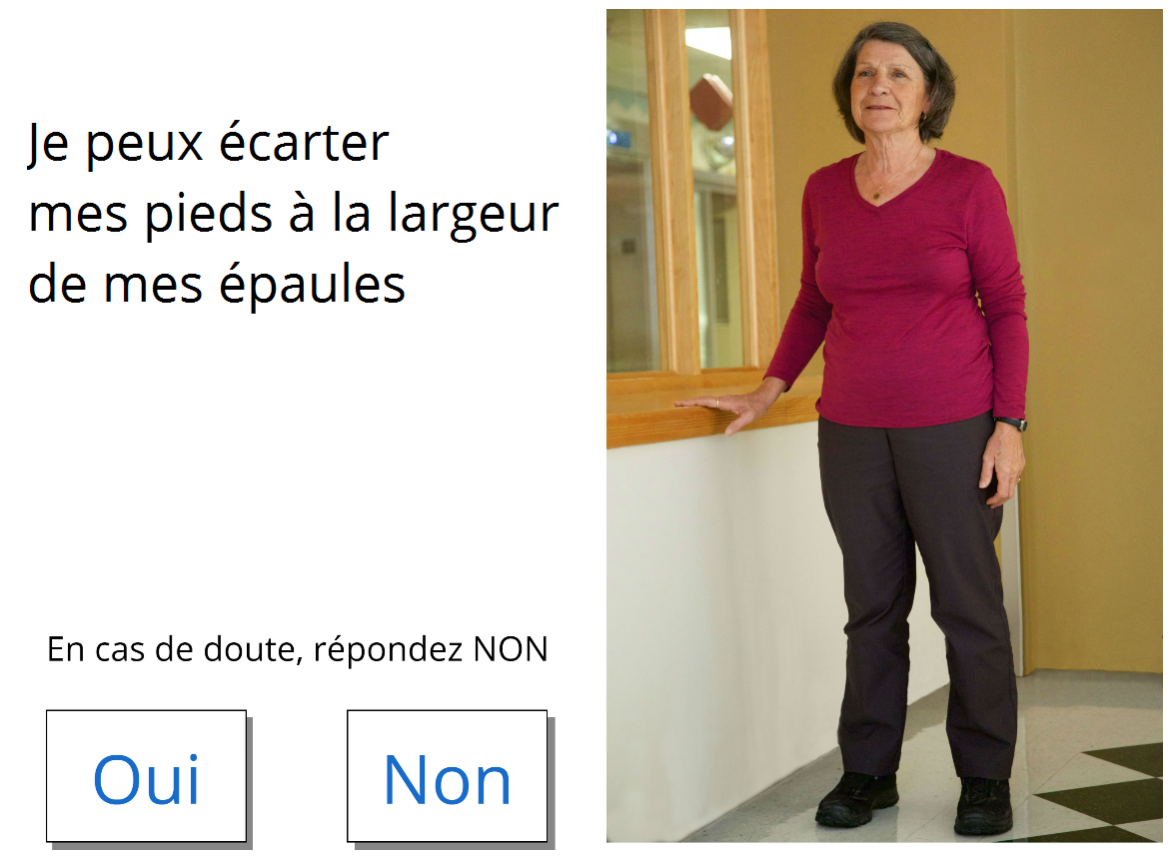
Excerpt from the French version of the full prototype (translation: I can stand with my feet shoulder-width apart; if not sure, answer No; Options: Yes, No).

Every participant was invited to explore the prototype as if they were using it alone on a personal device of their choosing while enunciating their thoughts out loud [32]. The interviewer would ask the reason for a certain reaction or facial expression. Thorough note taking and recording of interviews allowed us to compile and analyze comments, from which the designers and programmer evaluated the modifications deemed essential before further testing. The decision to modify the prototype following a comment was made by considering its relation to the themes drawn from the previous research on user needs; its influence on the user’s comprehension; the resources available; and the different, possibly contradicting, comments. All modifications applied to the prototype would always help the user’s comprehension to maintain the adequacy of the consequent recommendations. The comments with the highest impact on usability were prioritized to make corresponding changes (Figure 5). Modifications were made to the prototype following each group of the 3 tests described earlier. 

### Translation and Graphic Identity

**Figure 6:**
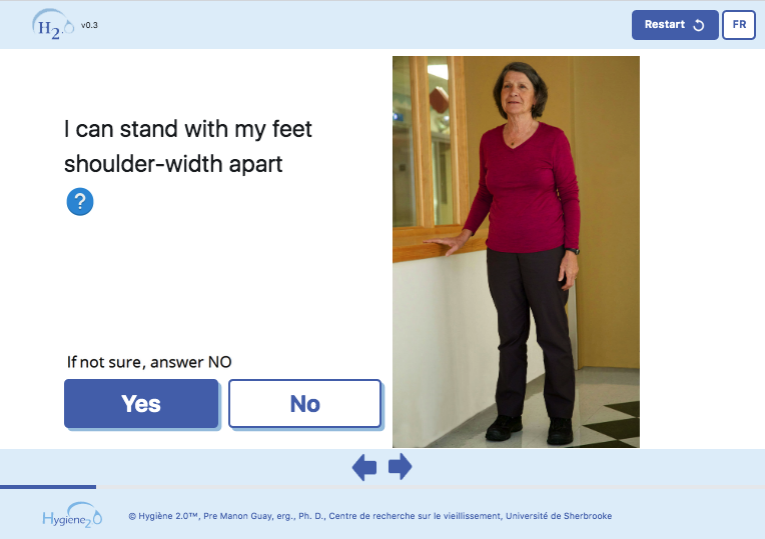
Excerpt from the English version of the full prototype.

French was the working language throughout the study. Therefore, the prototype was developed and tested in French. However, English being the language used by most Canadians, and a language for scientific communications, an English translation (Figure 6) was performed by the designers and reviewed by a certified translator. The designers and the programmer collaborated with a graphic designer to create a graphic identity for H_2_.0. 

### Ethical Considerations

This research project was approved by the ethics committee of the CIUSSS de l’Estrie-CHUS. Sociodemographic data were not gathered during the second, third, and fourth iterations. Indeed, the second and fourth iterations involved an expert who helped design and review the structure of the e-DSS and not interface use. The guerrilla testing on the third iteration values quick and numerous comments on a few key interface aspects, and therefore should not involve sociodemographic data gathering. Confidentiality during navigation on H_2_.0 was ensured by not asking for identity-related information (eg, no profile creation before use) and the computer’s internet protocol address was not collected. The website is hosted locally on the research center server.

## Results

### Participant Characteristics

A total of 47 different participants were recruited during the 5 iterations. For iterations 1 and 5, 24 (75%) of the participants were women. Tables 1 and 2 present the sociodemographic characteristics. Interviews lasted on average 48 min (range 30-82 mins), focus groups lasted on average 105 min (range 98-116 mins), and testing lasted on average 53 min (range 30-93 mins).

Table 3 presents the different devices and browsers used by the participants during the fifth iteration. Researchers and OTs preferred using their smartphones or laptops, whereas older adults and caregivers were generally more comfortable using their desktop computer or iPad. Tests performed on a variety of brands and internet browsers allowed verification of website functioning.

**Table 1:** Number and profile of the participants by iteration.

Participant profile	Iterations, n (%)					Total, n (%)
	First	Second	Third	Fourth	Fifth	
Older adults	8 (17)	N/A^a^	N/A	N/A	3 (6.4)	11 (23.4)
Caregivers	5 (10.6)	N/A	N/A	N/A	3 (6.4)	8 (17)
Occupational therapists	3 (6.4)	1 (2.1)	N/A	1^b^ (2.1)	3 (6.4)	7^b^ (14.9)
Researchers	N/A	N/A	N/A	N/A	3 (6.4)	3 (6.4)
Other stakeholders	4^c^ (8.5)	N/A	14^d^ (29.8)	N/A	N/A	18 (38.3)
Total	20 (42.5)	1 (2.1)	14 (29.8)	1^b^ (2.1)	12 (25.6)	47 (100)

^a^N/A: not applicable.

^b^The clinical expert was the same on the second and fourth iterations.

^c^Other stakeholders were members of health care interdisciplinary teams (n=1), members of community resources (n=2), and a bathing assistive technology provider (n=1).

^d^Passersby in rehabilitation or geriatric scientific conferences could include caregivers, clinical experts, researchers, and occupational therapists.

**Table 2:** Sociodemographic characteristics of participants for the first and last iterations (n=32).

Iteration and participants			Sample, n (%)		Age (years), mean (SD); (range)		Education (years), mean (SD); (range)		Use of internet (years), mean (SD); (range)		OT^a^ experience (years since graduation), mean (SD); (range)
**First iteration**											
	Interviewees	7 (22)		41 (7.4); (30-51)		17 (1); (16-18)		—^b^		N/A^c^	
	Focus group members	13 (41)		69 (7.1); (52-84)		14 (3.2); (8-18)		14 (9.7); (0-30)		N/A	
**Fifth iteration**											
	Prototype reviewers	3 (9)		49 (4); (45-53)		22 (0); (22-22)		23 (2.5); (20-25)		26 (4.1); (21-29)	
	Testers	9 (28)		61 (13.8); (41-76)		16 (3.2); (12-22)		24 (6.9); (12-30)		22 (6); (18-29)^d^	

^a^OT: occupational therapist.

^b^Not available.

^c^N/A: not applicable.

^d^Values only for the 3 testers who were OTs.

**Table 3:** Devices and browsers used during individual testing (fifth iteration).

Participants			Device used for testing		Brand		Internet browser used for testing
**Researchers**							
	1	Smartphone		Samsung		Chrome	
	2	Smartphone		Samsung		Chrome	
	3	Smartphone		Samsung Galaxy		Safari	
**Older adults**							
	1	Desktop		iMac Apple		Safari	
	2	Desktop		ASUS		Chrome	
	3	iPad		Apple		Chrome	
**Caregivers**							
	1	Desktop		ASUS		Firefox	
	2	Laptop and smartphone		Macbook pro and Samsung Galaxy A5		Chrome	
	3	iPad		Apple		Chrome	
**Occupational therapists**							
	1	Laptop		HP		Chrome	
	2	Smartphone		iPhone 6S		Safari	
	3	Smartphone		Samsung Note 3		Chrome	

### Identification and Integration of Stakeholders’ Perspective During Prototype Design

Overall, 4 emerging themes (and 7 subthemes) of an e-DSS for older adults having difficulty performing personal hygiene, and their caregivers, were identified from interviews, focus groups, and tests. These are focus (safety, confidentiality, autonomy, and well-being), engage, facilitate (simplify, clarify, and illustrate), and access. Multimedia Appendix 3 presents a summary of how these themes and subthemes were translated into features of the prototype to adapt Algo’s paper version into an e-DSS using the participants’ perspective. The following paragraphs describe the themes and subthemes, which are included in each title. The integration of these themes in the resulting website is also illustrated in a video [33].

#### Focus on Underlying Values

The e-DSS for persons having difficulty performing personal hygiene should focus on 4 purposes: safety, confidentiality, well-being, and autonomy.

#### Promote Safety During Navigation and After

The e-DSS should promote the physical safety of seniors with diminishing autonomy. Physical safety must be ensured not only during the use of the system but also during the implementation of its recommendations:

No, but it’s because you can see that there is no non-slip surface, you can see that there is no grab bar to hold on to. […] For me, it is not safe.

Focus group–caregiver

Q: You look at her position and you think: “Would I be able to take that position exactly?” A: Yes, that’s it.

Focus group–caregiver

Q: What do you imagine when you read this question (I can hold onto a grab bar with each hand) […]? A: Well, I think of safety.

Individual testing–older adult

In addition, OTs indicated that having users enter both feet in the bathtub could be unsafe for some of them. The e-DSS would need to indicate only lifting the feet up to the bath rim to test one’s capacity.

#### Ensure Confidentiality of Personal Information

The e-DSS should ensure the confidentiality of personal information. As the domestic help service director of a community resource explained in an interview, “… maybe there could be some reluctance from clients to enter their information into the client file… be careful with sensitive information…”

#### Enhance People’s Well-Being

The e-DSS should enhance people’s well-being. Sensible questions (eg, is the person at the end of his or her life?) should be rephrased and asked only if needed, and at the end of the navigation. Navigation and questions must also be straightforward to minimize the risk of confusion:

[…] caregivers who call us are in a higher age range […] so starting to explore each item when they already have some confusion about other things I think could be difficult for them.

Interview−advisor in a caregiver support organization

#### Promote Functional Autonomy of Users

The e-DSS should promote the autonomy of users seeking information about bathing difficulties and how to mitigate them:

It becomes humiliating, I find [to not be able to wash ourselves]. We can’t get along on our own, we feel diminished, we don’t feel like ourselves. It’s as simple as that. It isn’t much, but that’s the way it is.

Focus group–older adult

"I am finding it hard to wash myself” [opening question], that summarizes a little what we would need; give us tips […] because our mobility is reduced. I mean, my back hurts. It’s out of the question for me to wash my back or to wash below my knees. I can’t bend. […] Maybe I will never be able to do that again, but at least if they suggested some tips.

Focus group–older adult

#### Engage Users Throughout Navigation

The e-DSS should engage users from the very beginning, opening the navigation with their worries. The main worry is hygiene, as an older adult said during a focus group, *“Yes, I really found hygiene was the most difficult.”* Focus groups and interviews highlighted that in addition to difficulties in performing hygiene, two other major concerns must be addressed as opening questions. First, fear of falling while performing hygiene is a common reason to initiate bathroom adaptations:

[…] it’s not rare for the child to say “my mother fell in the bathroom on the weekend, and now I want to make the bathroom safer, what options are available?

Interview−director in an assistive technology store

Even today, getting in and out of the bathtub, I’m always afraid of falling. That has remained. So, you see, it’s been almost eight years. The fear has remained.

Focus group–older adult

Second, acquiring knowledge about available assistive technologies would also be a hook for potential users:

Sometimes caregivers would tell me “I bought this”… and they would ask me “is what I bought adequate?” […] Often the aids […] would ask “what do you think would be the safest to use as equipment?”… “is there another piece of equipment that could be safer, more adapted to the person?

Interview–OT

They give us tips like buying a sponge (the ones with the long handle) […], but to use it to reach our toes, that’s another story.

Focus group–older adult

#### Facilitate Navigation

The e-DSS should make navigation as easy as possible. This implies simplifying, clarifying, and illustrating.

#### Simplify the Navigation Experience

First, navigation on the e-DSS should be simple and therefore short in time, requiring a minimal number of steps:

Because on Internet, that’s the danger. We go over things quickly.

Focus group–older adult

You have to read a lot. I mean, nothing can be understood quickly in the image. You really must take your time to look at the […] different steps or different recommendations. You have to read. [Is it a lot of text for you?] Yes.

Focus group—older adult

[Did you think it was possible to […] go back only one screen?] Oh no, the thought did not cross my mind.

Individual testing–caregiver

#### Clarify Information Requested or Provided

Second, the information should be clear enough to facilitate the user’s general comprehension and ensure the accuracy of the answers given to the e-DSS’s questions. For example, users need to know what to answer if they are unsure of their capacities:

There is something about the closed question […] sometimes it’s as if they [elderly people] are uncomfortable, […] it creates some hesitation, raises some anxiety, it’s as if, well I don’t know if it’s yes, I don’t know if it’s no, well sometimes, so I don’t know what to answer.

Interview advisor in a caregiver support organization

It would have been yes and no. If it had been in cycles, it would have been no when I just got out, and, a month later, yes. [Would you have answered yes or no according to now, the moment when you responded to the questionnaire, is that it?] Exactly. Today, yes, but a month ago, no. Oh no. [Would it be better to say: “Today, can you…”] It might be better to explain the moment when you have to spread your legs 12 inches. That might be better.

Focus group–older adult

Consquently, to provide clarity to the user, the sentence “If not sure, answer No” was added above the Yes and No buttons.

 Another example of clarity is that the user must know exactly what to consider when answering the question:

Maybe have a note saying if it is for someone else, consider the other person’s capacities.

Individual testing–caregiver

[…] I don’t understand the context […] if I was a lady who did not have the notion of transfer capacity, I would wonder “why should I do that?” […] I would tend to word this question “I am able to move from one chair to the other” […]

Individual testing–OT

Clarity also means that the user must be aware of his progress while navigating. An OT said during individual testing, *“Well, it took me some time before I realized it was the end.”*

The e-DSS also ensures clarity by offering two navigation pathways, one for older adults and one for caregivers. The caregiver path gives the option of being accompanied and answering on behalf of someone else.

Maybe encouraging people to navigate with another person could be interesting also [...]

Quebec College of OTs (OEQ) staff member

#### Illustrate What Is Communicated

Specifically, the e-DSS should use images to promote understanding and simplification of the questions while still considering the user’s perceptions and preferences. Indeed, users rely on images to further understand the question and its context:

That image, for me, I find it very confusing, because I’m looking […] OK, a bath seat, I read the first sentence, “anti-slip mat”. But then I don’t see the relevance. […] I would move on to something else.

Focus group–older adult

Me, I am very visual, so I prefer with images.

Focus group–older adult

It should be as explicit as possible regarding what a standard environment is or what the particular characteristics can bring in terms of difficulty in installing the equipment so that what is usually done through the healthcare professional’s judgement can be done by the client.

Interview executive advisor for OT services

According to the picture, I would say that you have to use your hands [to get up]. [Individual testing–caregiver]

[What do you think of this picture compared to the drawing in the beginning?]. It’s [the picture] easier, we can really see what it is.

Individual testing–older adult

#### Ensure Ready Access to Users

The e-DSS should be readily accessible to users. Potential users said that they would be happy to use an electronic tool that would be provided to them via a web link by their health professionals:

I think it would be wonderful. I mean, I leave the hospital […] and I don’t have to go meet anyone at all, I just go on my computer. Advice, everything I need is there. I put them into action or I don’t. I just have to type on my keyboard and I have it all. All my data is there.

Focus group–older adult

After an operation, when we return home, they say: "There’s a computer program. We’ll send it to you. Check it out."

Focus group–older adult

Had I known that a computer program like that one existed [H_2_.0], I would have gone on Internet, yes, even if I’m not 100% computer-savvy. But I would have gone to check it out.

Focus group–older adult

Yes. I give my e-mail and it comes to my place. Hello.

Focus group–older adult

Furthermore, the use of an offline mobile app that can be downloaded at one time and used at another, when the internet is not available, is both practical and strategic. In fact, the internet is not always available to users for reasons related to cost or location (eg, cost of a data plan on a mobile device or availability of high-speed internet in rural areas). Users also have varying internet access depending on where they work and live:

I’m thinking of when I was working with a hospitalized clientele: it’s one computer for five professionals, and there is no computer having an access to internet.

Interview–OT

A choice of either an offline mobile app or a responsive website, using any device, portable or fixed, could guarantee availability to most users. Through the focus groups, we gathered information that users prefer different devices for different activities (eg, consult bank account, play games, read an email). Through interviews, it appeared that users might also not be able to choose which device they use in their work context because of what is provided to them and what is restricted for confidentiality reasons:

Well, normally it’s not well looked upon [to use a personal cell phone with a patient] on a confidentiality level and all.

Interview–OT

Well, if I had the choice, […] tablets are really an interesting technology because they are very easy to use.

Interview–OT

Users highlighted the importance of finding a name for the app that would contribute to accessing it. After brainstorming by the research team, the electronic prototype was named *H*ygiene *2.0*. This name is bilingual (French and English), short, and evocative of the purpose and the format.

### Hygiene 2.0 Full Electronic Prototype

Although H_2_.0 is an adaptation of Algo’s paper format decision support tool, some key differences exist between the 2. First, some questions are combined in the paper form (Figure 1), whereas the questions in the H_2_.0 e-DSS are asked one at a time. This is meant to meet the facilitating criteria, a need expressed by older adults and caregivers. Even though it might add steps, it makes each question simpler and clearer for both pathways, the one designed for older adults facing hygiene challenges, and the one for their caregivers navigating with, or for them.

Second, although the whole algorithm is presented at once to a health care worker using Algo’s paper format, in the H_2_.0 e-DSS, each question presented to the user will be determined by the response given to the previous question. This dynamic aspect of the electronic version makes the process different for each user.

**Figure 7:**
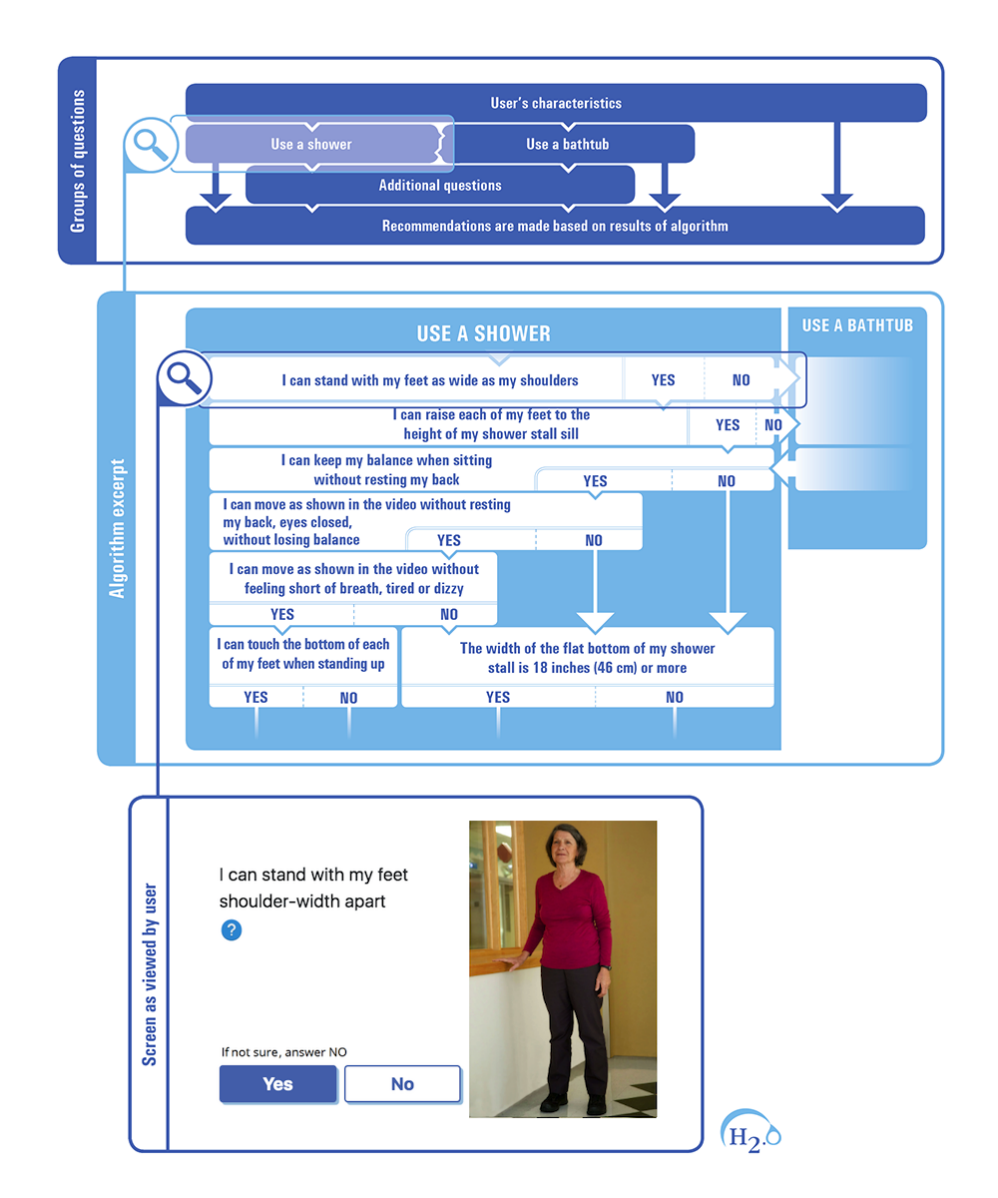
Structure of Hygiene 2.0.

Third, the items in Algo are structured in 4 sections. The first 2 sections are grouped according to 3 central concepts in occupational therapy: occupation, person, and environment (Figure 1). Indeed, components are addressed successively, starting with items related to the occupation, then the ones regarding the person, and finally, those related to his or her environment. This structure is no longer followed in H_2_.0 (Figure 7). All aspects are still covered, but the order favored is in line with user needs; for example, minimizing the number of questions and presenting the more sensitive ones at the end. The theoretical occupational therapy background is not as obvious for a lay user in H_2_.0 as it is in Algo.

Fourth, the paper format takes into account the user’s desire regarding the place where he or she wants to shower: *Wants to shower in a bathtub* (Algo) versus *I shower in a standard bathtub* (H_2_.0) and *Wants to shower in a shower stall* (Algo) versus *I shower in a standard shower stall* (H_2_.0). This was thoroughly discussed while comparing the algorithm structure of the paper form and the electronic form with an OT:

I find it’s at another level, not only preference. I can prefer washing myself in the bath but not be able to get into the bath […] so I don’t do it in the bath but I would like to do it in the bath. […] There is preference and then there is capacity, the fact that the shower or the bath becomes an architectural barrier.

Prototype review–OT

It is possible for a health care professional using Algo’s paper format with a client to consider his or her preference while taking into consideration his or her capacities as well as the presence of a standard shower or bathtub in the dwelling (Figure 1). This becomes more challenging when answering a series of questions presented one at a time, without the user’s knowledge of the whole algorithm. To minimize the number of questions and maintain the yes or no answering pattern, the questions regarding the place where the user showers were modified in the e-DSS. This meets the user’s need for facilitating. Although it does not consider the user’s preference for showering in the shower stall or in the bathtub, it still evaluates the capacity and architectural barriers (standard shower stall or bathtub).

Fifth, to enhance facilitating while ensuring safety as desired by potential users, some details are not made explicit in the text but by combining text and picture, thereby minimizing the amount of text. For example, the question in the H_2_.0 e-DSS does not explicitly state that the client can use some form of support as it is done in the Algo user guide (Figure 6). However, the picture shows a woman using a support with her right hand.

**Figure 8:**
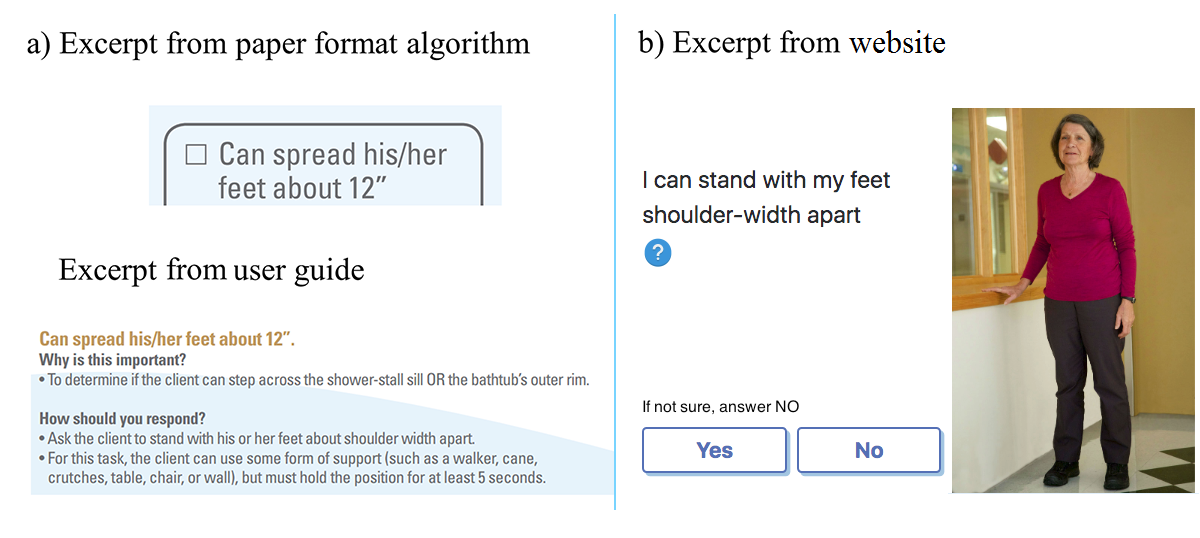
Spreading feet about 12 inches. (a) Paper format Algo and (b) website Hygiene 2.0.

Sixth, to further enhance facilitating while answering questions, some formulations were adapted. Instead of asking the user to spread his or her feet 12 inches apart as in Algo, the shoulder-width reference is used in H_2_.0 (Figure 8).

## Discussion

### Principal Findings

This study aims to adapt Algo’s paper form into an e-DSS to allow self-selection of bathroom adaptation by community-dwelling older adults. On the basis of a user-centered design method, e-DSS H_2_.0 was created by taking into consideration the perspective of older adults having difficulty performing personal hygiene in their home, caregivers, OTs, assistive technology providers, and community resources. To enhance usability, H_2_.0 focuses on 4 purposes (safety, confidentiality, well-being, and autonomy); engages users from the beginning by listing potential major concerns on the first page; and eases navigation as much as possible by simplifying, clarifying, and illustrating. For older adults having difficulty bathing (or their caregivers), navigating on such an e-DSS would enable them to, for straightforward cases, self-select common assistive technologies fitting their needs to promote autonomy and safety. The more complex cases identified will be redirected to occupational therapy services.

One might wonder about the choice of opting for a digital tool for people aged 65 years and older. Seniors, particularly those aged between 65 and 74 years, are increasingly using the internet as a source of information for their health. According to a CEFRIO (Centre facilitant la recherche et l’innovation dans les organisations) survey, 80% of people living in the province of Quebec, Canada, aged 65 years now have an internet connection at home, 60% use the internet every day, and 76% of internet users evaluate their personal internet use skills at an average or high level [34]. Digital technologies are, therefore, becoming major potential vectors to help older people take care of their health. However, it is important not to exclude those who do not use technologies; hence, the importance of caregivers being involved in the development of H_2_.0 and the e-DSS giving an option to answer on behalf of someone else. Community pharmacists that sell assistive technology for hygiene could also be users of H_2_.0 as well as other formal resources available in the community. This would allow people who are unfamiliar with the internet to receive guidance when using the website. Besides including the possibility of receiving help, the website is also easy to use for people with lower digital literacy. Nonetheless, as the older adults and caregivers who tested the website during the fifth iteration had been using the internet for many years, future usability tests should involve people with lower digital literacy.

According to the World Health Organization (WHO) guidelines, H_2_.0 has reached the mid-demonstration stage [35] for monitoring and evaluating digital health interventions throughout their development process from the prototype stage to full implementation of the technology. The WHO defines 6 stages of an intervention maturity life cycle (preprototype, prototype, pilot, demonstration, scale-up, integrated and sustained program) grouped into 3 larger categories: early, mid, and advanced stages. The H_2_.0 e-DSS has been tested with users successively in an increasingly realistic environment, the latest of which is the older adults and caregiver’s homes, navigating with their personal history in mind on their personal device. Therefore, realistic insights have been gathered to customize H_2_.0 for a near future scaling-up of this electronic health intervention.

Nevertheless, H_2_.0 can be considered as an intervention applied in controlled conditions, limited in terms of population and geography testing. It has yet to be deployed on a larger scale to reach its full implementation, which will be the subject of further studies as it will bring forth new challenges. Among other things, this will mean adapting the recommendations to the implementation context and creating the different features that were suggested during the focus groups and interviews but have not yet been applied (Multimedia Appendix 3). For example, more information regarding the questions and recommendations should be made available with links and help buttons. Moreover, a small convenience sample did not allow the capture of a variety of digital literacy and bathing difficulties, which should be pursued through additional field testing. The psychometric qualities of an e-DSS are also important aspects to investigate before full-scale implementation [36].

This research was conducted in French as it is the primary language in Quebec, Canada; the English version of H_2_.0 lacks transcultural adaptation [36], a process that could be conducted further on. Despite 2 bilingual content experts and a translator revising the last iteration, the prototype might still employ field-specific terms that need to be adapted to make them understandable to the general French- and English-speaking public while conveying the correct meaning.

### Conclusions

On the basis of knowledge embedded in the clinical *algo*rithm Algo, H_2_.0 [33] is an e-DSS conceived for individuals having difficulty performing personal hygiene or people concerned about or assisting a community-dwelling older adult. H_2_.0 focuses on safety, confidentiality, well-being, and autonomy to support the self-selection of common assistive technologies. Facilitating assistive technology provision for hygiene using H_2_.0 implies simplifying, clarifying, and illustrating yes or no simple questions initially designed for non-OTs as well as developing an accessible format such as a responsive website and an offline mobile application. H_2_.0 has reached the demonstration stage and could be integrated in a continuum of care to enhance the personal hygiene of community-dwelling older adults.

## Appendix

Multimedia Appendix 1 Interview guide for stakeholders.

Multimedia Appendix 2 Interview guide for focus groups.

Multimedia Appendix 3 Integration of stakeholders’ perspective while designing an electronic prototype (H_2_.0).

## Abbreviations

e-DSS: electronic decision support system
H_2_.0: Hygiene 2.0 (electronic prototype)
ISO: International Organization for Standardization
OT: occupational therapist
WHO: World Health Organization
