# Supplementary material for: Self-Selection of Bathroom-Assistive Technology: Development of an Electronic Decision Support System (Hygiene 2.0)
Source: J Med Internet Res. 2020 Aug 10;22(8):e16175. doi: 10.2196/16175 (PMC7445614; doi:10.2196/16175)
Supplement: Multimedia Appendix 1 [file jmir_v22i8e16175_app1.pdf]

Appendix 1: Interview guide for stakeholders

Work environment

1. Describe briefly the functions performed and the assignments included in your work
2. Describe the human and organizational environment at work (relations with colleagues and clients) :
   a. Do you mostly work in team or individually?
   b. How do you establish contact with your clients?
   c. How do you share information with your colleagues/ clients?
   d. Other details...
3. Describe your workplace's physical environment:
   a. Do you mainly do desk work?
   b. If yes, which equipment do you have at your workplace?
   c. If no:
      i. To which equipment do you have access outside your usual workplace?
      ii. Which equipment do you bring with you?
4. What is your use of ICT (information and communication technology) within your work, at the equipment level as well as the software level (ex: email, web sites, software, data base, portable or fix computer, cell phone, Ipad, etc).
   a. What are you using at your usual workplace?
   b. What do you use if you work outside your workplace?
   c. What are your preferences in this regard (which equipment/software do you prefer using and why?)
5. How often do you use each of the ICT (equipment and software) mentioned in your work?

Assistive technology information (requests, access, distribution)

6. In your daily work, how often do you have to advise people (older adults or others) looking for information on assistive technologies?
7. If this is the case in your work, describe how those demands are addressed to you (ex: describe one (or more) situation in which you had to advise a person with autonomy losses who was looking for information on assistive technologies).
8. What are the most common questions you are asked in regards to safety and hygiene in the bathroom?
9. Which information do you have on safe bathroom designs (ex: assistive technologies)?
    a. How do you access it?
    b. How do you distribute this information (if this is the case)?
10. Which information do you think should be available to older adults in regards to safe bathroom design (ex. assistive technologies)?
    a. In which form?

e-Health

11. What do you think about «e-Health»?

    (Government of Canada definition: «overarching term used today to describe the application of information and communications technologies (ICT) in the health sector. It encompasses a whole range of purposes from purely administrative to health care delivery. »)

    (Government of Canada definition of ICT: «The Digital Technologies sector is a combination of manufacturing and service industries that use creativity, talent, and digital skills to capture, transmit and display data and information electronically. The sector consists of the established core of the information and communications technology (ICT) subsector, with lucrative new vertical markets developing in healthcare, education, finance, defense and the creative industries as IT-based systems increasingly underpin operations across the economy. »)

    a. What are the advantages?
    b. What are the inconvenient?

Comments on the paper format Algo and the needs regarding an electronic version

12. Present the paper format Algo and give enough time so the participant can read the document.
    a. What do you think about this tool?
        i. What are the key strengths?
        ii. What are the weak points?
13. Could you include the paper Algo in your work, if yes, how?

14. What would make you use a similar tool on an electronic support?
    a. What would you find it relevant/useful?
    b. What could make the use a computer support interesting?
15. Which information should be in the computer support, except the questionnaire?

Suggestions

16. As the interview reaches its end, would you like to clarify some aspects or bring out other subjects? If yes, which one?

17. If it is the case, do you have any other suggestions?
