# Supplementary material for: Self-Selection of Bathroom-Assistive Technology: Development of an Electronic Decision Support System (Hygiene 2.0)
Source: J Med Internet Res. 2020 Aug 10;22(8):e16175. doi: 10.2196/16175 (PMC7445614; doi:10.2196/16175)
Supplement: Multimedia Appendix 2 [file jmir_v22i8e16175_app2.pdf]

Appendix 2: Interview guide for focus groups

Interview guide for older adults and caregiver focus groups

A. Experience in regard of a safe bathroom design and the choice of assistive technology.

- Could you tell us about your **experiences** when the time came to **make safety changes** in your/your relative's bathroom.
  - Description of the situation
  - **How** did you find the information?
  - **Where** did you go?
  - **Who** did you go to?
- If you/your relative own(s) an assistive technology for your personal hygiene, could you describe **the steps you went through from the choice to the use** of the assistive technology?
  - Choices, answers to your questions, choices, purchase, use
  - What was the **easiest**?
  - What was the **hardest**? - *challenges, complicated steps*
- Following the experience you had, which information do you believe **should be made available** and could **be useful** in regard to the safe design of a bathroom and the selection of an assistive technology?
  - In which **form**? - *pamphlet, computer, auxiliaries*

B. ALGO: Relevance, use, and advantages of an electronic version.

Present an example of the electronic version of the Algo

Explain how an electronic version of the Algo could be usable by people with autonomy losses and their relatives.

- Would you see some **benefits** in the use of a similar tool on a computer support?
  - What could be **relevant/useful** for you?
  - What could make the use a computer support **interesting**?

C. Needs in regards to health information

- Do you use the **Internet** to obtain **health information**? (Banas, 2009)
  - If yes, **which** kind of information are you looking for?
  - If no, **where** do you find your information (pharmacy, clinic, relatives or caregivers, friends)?
  - With or without the use of internet, how do you **save** the information you find useful?
  - **[**If yes,**]**
    - In your opinion, what are the positive aspects of the internet when you are looking for health information? (Banas, 2009) *- Describe your positive experiences*
    - In your opinion, what are the negative aspects of the internet when you are looking for health information? (Banas, 2009) *- Describe your negative experiences*
- **[**Among the web sites lists you obtain during the research, what pushes you to: **]**
  - **Click** on the web site link
  - Choose to **explore** or not the web site more extensively
- If there was an **electronic tablet in the pharmacy** containing health information, would you be willing to use it?

D. Use of computer technology

- Let's talk about the software support you are using (computer, tablet, Smartphone) and the reasons why you use them (email, text writing, budget, internet)?

  - Associate the type of software support to the activity

- Now, let's talk about your personal **preferences**. Which type of software support do you prefer to use and for which reasons?

☐ socio-demographic questionnaire
☐ Acknowledgments

E. Conclusion

- As the focus group reaches its end, we still have X minutes if you want to clarify some aspects or bring out suggestions.

☐ confidentiality reminder
☐ Acknowledgments
☐ Submit the consent form and the socio-demographic questionnaire
