# Supplementary material for: Self-Selection of Bathroom-Assistive Technology: Development of an Electronic Decision Support System (Hygiene 2.0)
Source: J Med Internet Res. 2020 Aug 10;22(8):e16175. doi: 10.2196/16175 (PMC7445614; doi:10.2196/16175)
Supplement: Multimedia Appendix 3 [file jmir_v22i8e16175_app3.docx]

Integration of stakeholders’ perspective while designing an electronic prototype (H_2_.0)

| **Feature of the prototype** | **Themes and subthemes** | | | | | | | | |
| --- | --- | --- | --- | --- | --- | --- | --- | --- | --- |
|  | Focus | | | | Engage | Facilitate | | | Access |
|  | Safety | Confidentiality | Autonomy | Well-being |  | Simplify | Clarify | Illustrate |  |
| Do not perform full bath transfer | P^a^ | —^d^ | — | — | — | O^a^ | — | — | — |
| Illustrate safe postures and safe use of AT^b^ | P | — | — | — | — | — | O | O | — |
| Warn and instruct regarding safety (AT installation and recommendations)^c^ | P | — | — | — | — | — | O | O | — |
| Advise navigating with another person | O | — | — | O | — | O | P | — | — |
| Do not ask for identity-related information | — | P | — | — | — | — | — | — | — |
| Add warnings at the end instead of a sensitive question in the beginning (end-of-life and degenerative disease) | — | — | — | P | — | — | — | — | — |
| Compensate for doubt when considering a response choice (deal with varying capacity and use closed question) | O | — | — | O | — | — | P | — | — |
| Explain the reason for recommendations^c^ | O | — | P | — | — | — | O | **—** | **—** |
| Direct to other resources^c^ | O | — | P | O | — | — | — | — | — |
| Include advice regarding personal hygiene^c^ | O | — | P | O | — | — | — | — | — |
| Create appealing opening questions (algorithm entry on the first screen) | — | — | — | O | P | — | — | — | — |
| Ensure sufficient contextual setting on the first screen | — | — | — | O | — | — | P | — | — |
| Simplify tests to perform | — | — | — | O | — | P | — | O | — |
| Minimize the number of steps, tests**,** and questions | — | — | — | O | — | P | — | — | — |
| Minimize text | — | — | — | O | — | P | — | — | — |
| Present AT images in their context of use | O | — | — | — | — | — | O | P | — |
| Be careful with image detail | O | — | — | — | — | — | O | P | — |
| Use photos and videos (postures and AT use) | O | — | — | — | O | — | O | P | — |
| Use images instead of text (eg**,** to define a standard bathtub) | — | — | — | — | — | O | — | P | — |
| Make available in the form of an offline mobile app and a responsive website^c^ | — | — | — | — | — | O | — | — | P |
| Connect users with professionals^c^ | — | — | O | — | — | O | — | — | P |
| Make the website available at the pharmacy and in AT providing stores^c^ | — | — | O | — | — | O | — | — | P |
| Distribute on the internet and elsewhere^c^ | — | — | O | — | — | O | — | — | P |
| Rename Algo to Hygiene 2.0 | — | — | — | — | O | O | — | — | P |
| Adapt literacy level to 10 years old | — | — | — | — | — | P | — | — | — |
| Give web link name identical to the app | — | — | — | — | — | P | — | — | O |

^a^Cells with a P indicate the theme most related to this feature of the prototype and cells with an O indicate other related themes.

^b^AT: assistive technology.

^c^These features have not already been applied to the prototype.

^d^Cells have dashes when themes and subthemes are not applicable to the feature.
